# Intercalating Graphite‐Based Na‐Ion Battery Anodes with Integrated Magnetite

**DOI:** 10.1002/smsc.202400405

**Published:** 2024-12-19

**Authors:** Rukshan Karunarathna, Harsha Ranasinghe Arachchige, Shadeepa Karunarathne, W. Parakrama Sanjeewa Lakshitha Wijesinghe, Chanaka Sandaruwan, M. M. M. Prasanga Gayanath Mantilaka, Yasun Y. Kannangara, Amr M. Abdelkader

**Affiliations:** ^1^ Postgraduate Institute of Science University of Peradeniya Peradeniya 20400 Sri Lanka; ^2^ Centre for Nanodevices Fabrication and Characterization (CNFC) Faculty of Technology Sabaragamuwa University of Sri Lanka Belihuloya 70140 Sri Lanka; ^3^ Science Department Sri Lanka Institute of Nanotechnology Technology Park Homagama 10800 Sri Lanka; ^4^ Faculty of Science and Technology Bournemouth University Talbot Campus, Fern Barrow Poole BH12 5BB UK; ^5^ QBITS Labs, Research and Development Codegen International (Pvt) Ltd Trace Maradana 01000 Sri Lanka

**Keywords:** electrochemical magnetite functionalization, expanded interlayer spacing, intercalating graphite anodes, sodium‐ion batteries

## Abstract

Graphite is known as the most successful anode material found for Li‐ion batteries. However, unfortunately, graphite delivers an ordinary capacity as anode material for the next‐generation Na‐ion batteries (SIBs) due to difficulties in intercalating larger Na^+^ ions in between the layers of graphene due to incompatible *d*‐spacing. The methodologies investigated in deriving suitable anode structures for SIBs are found to be either less effective, expensive, or rather too complex in most cases. Herein, a simple strategy is introduced to derive suitable anode materials for SIBs through a modified electrochemical exfoliation of graphite. The introduced exfoliation process is able to graft Fe_3_O_4_ (magnetite) on graphite allowing the structure to expand, supporting a swift intercalation and deintercalation of Na ions. The synthesized magnetite‐functionalized graphene nanoplatelets are identified as a well‐suited anode material for SIBs, with its efficient intercalation obtained through the expanded interlayer spacing of 3.9 Å and the surface redox pseudocapacitive activity attained through the surface‐grafted magnetite. The effectiveness of the synthesized is reflected in the obtained high discharge capacitance of 420 mAh g^−1^, with 96% capacitive retention over 1000 cycles. The study opens new opportunities for prospective low‐cost anode materials for energy storage applications.

## Graphical abstract

A simple electrochemical exfoliation method modifies graphite to synthesize magnetite‐functionalized graphene nanoplatelets (Mag‐GNP) with an expanded interlayer spacing of 3.9 Å. The composite demonstrates enhanced Na‐ion intercalation, superior pseudocapacitive activity, and remarkable cycling stability. Mag‐GNP achieves 420 mAh g^−1^ reversible capacity and 96% retention over 1000 cycles, presenting a cost‐effective and scalable solution for advanced sodium‐ion battery anodes.

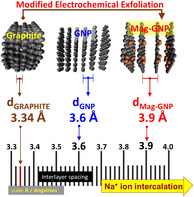

## 1 Introduction

Catering to the demand for high‐performance energy storage systems is crucial as the future growth of society is centered upon access to portable electronics, electric vehicles, and grid‐level energy storage.^[^ ^1^, ^2^ ^]^ To date, lithium‐ion batteries have paved the way for reliable power sources over the last few decades.^[^ ^2^, ^3^ ^]^ However, with the concerns of limited and unevenly distributed lithium resources, sodium‐ion battery (SIB) technology has taken a greater interest in meeting emerging and demanding applications.^[^ ^3^, ^4^ ^]^ Over the decades, graphite has been used as the most versatile anode material in Li‐ion batteries (LIBs).^[^ ^5^ ^]^ However, the larger radius of Na^+^ ion (1.02 Å) compared to Li^+^ ion (0.76 Å) affects Na^+^ ion mass diffusion into the graphitic lattice, causing poor storage efficiency in graphite anodes for SIBs.^[^ ^6^ ^]^ Hence, experiments report a small reversible capacity of ≈35 mAh g^−1^^[^ ^7^ ^]^ for SIBs using graphite anodes, which is an order of magnitude lower compared to the theoretical capacity of graphite (≈372 mAh g^−1^)^[^ ^8^ ^]^ for LIBs. Hence, it is crucial to find anode materials with high capacity, good cycle performance, and low cost for the next‐generation SIBs.^[^ ^4^ ^]^

In the quest to identify more diffusion‐capable anode structures suitable for SIBs, researchers have explored a range of carbon materials. These include carbon materials with varying structures (such as soft and hard carbons),^[^ ^9^ ^]^ different compositions (such as hydrogen‐containing carbons),^[^ ^10^ ^]^ and diverse morphologies (such as carbon nanotubes, nanowires, porous carbon nanoparticles, and reduced graphene oxides [RGO]).^[^ ^11^, ^12^, ^13^ ^]^ However, these materials have distinctive problems that are inherent. In comparison, hard carbons have shown more success recently. However, their progress is hindered by low initial Coulombic efficiency (ICE), which is primarily caused by irreversible pore filling.^[^ ^14^ ^]^ Carbon nanotubes are more expensive than other carbon anode materials in terms of manufacturing costs,^[^ ^15^ ^]^ while RGO exhibits poor reduction, leading to lower electrical conductivity.^[^ ^1^ ^]^ In addition, the recently developed 2D metal organic frameworks (MOFs) were also shown a limited performance as SIB anodes.^[^ ^16^ ^]^ Alloying‐type anode materials face significant volumetric expansion issues in SIBs compared to LIBs.^[^ ^17^, ^18^ ^]^ Hence, insertion‐type anodes, or the layered structures with the required diffusion capabilities (with reversible capacities),^[^ ^19^, ^20^ ^]^ or the hybrid systems of both seems to be most suited as the anode structure for SIBs. In this regard, recent theoretical studies shed light on the requirements for Na^+^ intercalation, which identified the interlayer distance should be a minimum of 0.37 nm in graphitic structures to achieve sufficient accommodation of Na^+^ ions without causing substantial mechanical stress, leading to fast pulverization of the anode.^[^ ^21^ ^]^

Conversion reaction‐based anode materials have also gained increased popularity over recent years due to their capabilities to achieve high capacities compared to diffusion and insertion‐type anode materials.^[^ ^22^ ^]^ Typically, the formation and the breakage of chemical bonds occur during the respective sodiation and the desodiation steps as illustrated in the following equation:

(1) $$M_{a}X_{b}+(b\times n){\text{Na}}^{+}+(b\times n)e⇌aM+b{\text{Na}}_{n}X$$

*M* denotes a transition metal and typically oxide, sulfide, or phosphide is denoted as *X*. Using this approach, researchers have been able to utilize magnetite nanoparticles and different materials such as FeP, FeS,^[^ ^23^ ^]^ and VO_2_.^[^ ^24^ ^]^ Iron oxides such as hematite, maghemite,^[^ ^25^ ^]^ and magnetite^[^ ^26^ ^]^ were highly exploited conversion reaction‐based anode materials in recent years due to the high abundance, and availability of simple and low‐cost synthesis approaches. Among iron oxides, hematite (Fe_3_O_4_) is considered better suited as it effectively facilitates the redox reactions with multiple Fe valances, as observed in various energy storage applications.^[^ ^27^ ^]^ However, the Fe_3_O_4_ (magnetite) has been attributed to a significant volume expansion and mechanical strains of the electrode material during the sodiation.^[^ ^28^ ^]^ In the literature, the nanostructured Fe_3_O_4_ and its composite materials have been introduced to control these volume changes with good cycle stability by reducing large mechanical deformation.^[^ ^29^ ^]^ Further, the intrinsic insulating properties in magnetite are also a major obstacle in ensuring rapid electron transfer and low internal resistance.^[^ ^30^ ^]^ Embedding Fe_3_O_4_ into a conductive carbon matrix is a well‐suited approach to tackling the mentioned constraints.^[^ ^31^ ^]^ Even though recent studies were reported with compositing Fe_3_O_4_ with RGO,^[^ ^32^ ^]^ Pyrolytic carbon, and other graphitic carbon structures^[^ ^33^ ^]^ with limited success, only a few studies attempted to develop a composite anode material for a SIB with their components complementing each other. With that, only a superior overall battery performance, including effective charge separation, pseudocapacitance, and intercalation, could be achieved.^[^ ^34^ ^]^ In that sense, Fe_3_O_4_ embedded into a suitably expanded graphitic matrix could provide an ideal platform to achieve high Na^+^ ion storage, where graphene layers act as a conductive matrix for support in fast electron transfer and assist in the distribution of Fe_3_O_4_ nanoparticles so that they do not aggregate but remain well dispersed. The discharging battery will benefit from the synergy of intercalation of diffused sodium ions within the graphene layers, along with simultaneous conversion reactions with Fe_3_O_4_. The large surface area of graphene is likely capable of letting volume changes inherent in conversion reactions of Fe_3_O_4_^[^ ^35^ ^]^ while providing a strong mechanical and structural enhancement to the electrode. The mentioned synergistic mechanism of operation would deliver a high capacity with improved electrochemical performances, including cycling stability and rate capability.

In this study, we have developed graphene nanoplatelets (GNP) and magnetite nanoparticle‐coated GNP (Mag‐GNP) with an interlayer distance larger than conventional graphite using a modified electrochemical exfoliation methodology. While GNP has small graphitic particles with an unchanged interlayer distance, we used Fe^3+^ and Fe^2+^ ions from the magnetite particles with a theoretical capacitance of 928 mAh g^−1^^[^ ^33^ ^]^ to expand the interlayer distance in Mag‐GNP. Our various characterization techniques have shown that the composite product Mag‐GNP can intercalate with Na^+^ ions several times higher than conventional graphite and RGO electrodes. We compared the electrochemical performance of the Mag‐GNP with the graphite, RGO, and GNP anodes in coin cell half‐cell configuration. The electrochemical measurements showed that the Mag‐GNP material had enhanced properties for Na‐ion storage, including high specific capacity, excellent rate capability, and stable capacity retention. Our study marks a significant step forward in the development of high‐capacity Mag‐GNP anodes for Na^+^ ion batteries.

## 2 Experimental Section

### 2.1 Synthesis of GNP and Mag‐GNP Nanostructures

**Scheme 1:**
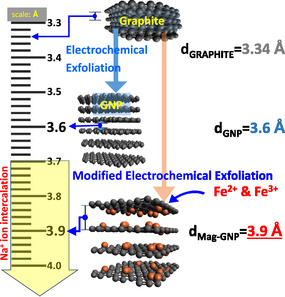
Synthesis of Mag‐GNP and GNP by normal and modified electrochemical exfoliation.

A modified electrochemical exfoliation process of graphite was used to produce Mag‐GNP. A solution was prepared by mixing 0.002 moles of Fe^2+^ ions with 0.004 moles of Fe^3+^ ions in 100 mL of deionized water. To obtain the formation of magnetite (Fe_3_O_4_), the molar ratio of Fe^2+^ and Fe^3+^ kept at 1:2 in the electrochemical reaction.^[^ ^36^ ^]^ The anode electrodes, constructed using Sri Lankan vein graphite (Bogala Mine), and the cathode electrodes, manufactured from stainless steel, were placed in a solution of 5 M Na_2_SO_4_, with a volume of 350 mL. All the chemicals were analytical grade and purchased from Sigma–Aldrich. The gap between the anode and cathode was maintained at 1 cm throughout the process. First, the graphite layers were expanded by applying a voltage of 2 V for 30 min through two electrodes, before the potential was raised to 10 V to initiate the exfoliation. Simultaneously, the Na_2_SO_4_ solution underwent the gradual addition of 100 mL of the pre‐prepared Fe^2+^/Fe^3+^ ion mixture drop by drop over 4 h. Following this, the mixture underwent continuous stirring for 12 h to achieve the synthesis of magnetic graphene. Afterward, the black color sediment was obtained and underwent multiple washes with deionized water using a vacuum filtration system. After Mag‐GNP was dried in a vacuum oven, it was stored in the neutral/ Ar environment to prevent oxidation by atmospheric air. For comparison, GNP was also synthesized electrochemically, without adding the Fe^2+^/Fe^3+^ ion mixture into the electrolyte (**Scheme** **1**).

### 2.2 Material Characterization

A crystallographic study was carried out by X‐ray diffraction (XRD) (model: Bruker D4 X‐ray scattering system with Ni‐filtered Cu Kα radiation) in the range of 15°–75°. Raman spectra were recorded from 500 to 3500 cm^−1^ on Senterra Bruker Raman Microprobe using a 532 nm, 10 mW laser, and 100X objective lens to determine the quality of the graphene structure in Mag‐GNP and GNP. Then functional groups in the synthesized Mag‐GNP and GNP were analyzed by Fourier transform infrared (FTIR) spectroscopy with the Bruker Vertex80 in the range of 500–4000 cm^−1^ of wavenumber in attenuated total reflectance (ATR) mode. External topologies and internal morphologies of the synthesized materials were identified by transmission electron microscopy (TEM) (model: Jeol 2100 microscope) and scanning electron microscopy (SEM) model: (Hitachi SU6600 microscope), respectively. Additionally, atomic force microscopy (model: The Park Systems XE‐100 instrument) was used to estimate the surface roughness and to investigate the thickness of the Mag‐GNP nanocomposites as well. The X‐ray photoelectron spectroscopy (XPS) measurements were obtained using a Scienta ESCA 200 spectrometer in an ultrahigh vacuum (10^−10^ mbar) to investigate the bond formation and to calculate the band gap of the Mag‐GNP nanocomposite. Thermogravimetric analysis was performed to examine the variation of composite mass with the temperatures.

### 2.3 Electrochemical Characterization

The coin‐type half cells (CR 2032) for SIBs were fabricated to evaluate the electrochemical performance. For SIBs, the Mag‐GNP composite anode was prepared by a cast‐coating technique. An electrode slurry was made by mixing 80 wt% of as‐synthesized Mag‐GNP composite, 10 wt% of Super‐P, and 10 wt% of polyvinylidene fluoride in *N*‐methyl‐2‐pyrrolidone, followed by doctor‐bladed on a Cu current collector. The fabricated electrode on Cu foil was dried in a vacuum oven at 140 °C for 10 h. The fabricated anode as a working electrode (diameter: 12 mm) with a mass loading of ≈1 mg cm^−2^ was fabricated into the coin cells with Na alloy (diameter: 15 mm) for the counter/reference electrode. For the electrolyte, 1 M NaPF_6_ was dissolved in ethylene carbonate/diethyl carbonate (EC/DEC 1:1 v/v) and for the separator Whatman glass fiber membrane (diameter: 20 mm) was used. The electrodes and cells for graphite, RGO, and GNP were prepared using the same process. All the fabrication processes were conducted inside the inert environment of the glove box filled with Ar gas (≈0.1 ppm of O_2_/H_2_O level). After the assembly, all the cells were rested at room temperature overnight.

The cyclic voltammetry (CV) under a scan rate of 0.1–0.5 mV s^−1^ and electrochemical impedance spectroscopy (EIS) under 10 mV AC voltage with a frequency range of 0.01 Hz–100 kHz were performed by the electrochemical working station (Metrohm‐Autolab PGSTAT302N, The Netherlands). The charge/discharge cycling under constant and varying current densities was conducted by a battery test system (LAND battery cycler CT3002AU) for cycle and rate performance measurements under a potential gap of 0.01–2.0 V, respectively. The specific capacity was calculated based on the mass of the anode materials in the prepared electrodes. The galvanostatic intermittent titration technique (GITT) measurement during the first discharge/charge cycle was performed under potential range of 0.01–2.0 V at 15 min for each pulse for both charge and discharge rate at 0.2 C and following 1 h for relaxation.

## 3 Results and Discussion

**Figure 1:**
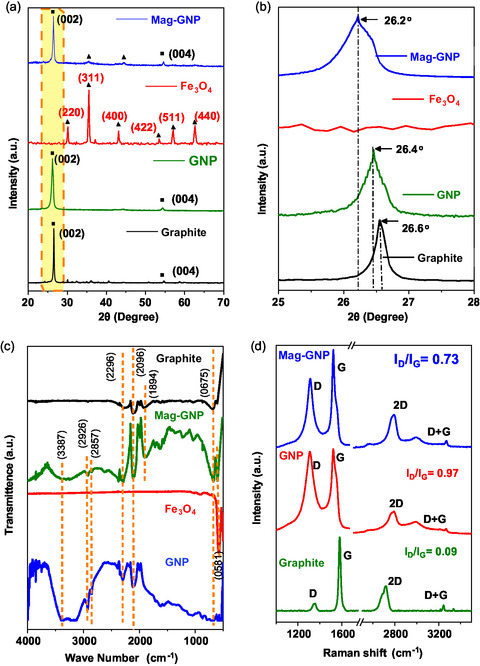
a) X‐ray diffractograms and b) its magnified 002 miler indices, c) FTIR spectra, and d) Raman spectra of Mag‐GNP, GNP, and their starting raw material.

The crystallographic patterns of the obtained Mag‐GNP and GNP were confirmed by XRD, as shown in **Figure** **1**a. For comparative analysis, the XRD patterns were compared with Fe_3_O_4_ (magnetite) and raw graphite powder. In Figure 1a, graphite pattern matched with the standard XRD pattern of graphite (JCPDS card no. 41‐1487)^[^ ^32^ ^]^ and the Fe_3_O_4_ pattern agrees with the standard pattern of magnetite (JCPDS card no. 19‐0629).^[^ ^37^ ^]^ Magnetite has shown the 2*θ* values of 18.3°, 30.1°, 35.2°, 43.1°, 53.6°, 56.9°, and 62.5° that correspond to Miller index reflections of (111), (220), (311), (400), (422), (511), and (440), respectively.^[^ ^37^ ^]^ The reflections from (002), (100), and (004) basal planes are responsible for the GNP sample's XRD peaks, which are located at 2*θ* values of 26.1°, 44.4°, and 54.3°, respectively, and are directly inherited from the graphite.^[^ ^32^ ^]^ This indicates that the GNP and graphite mostly have the same crystal structure. Mainly, compared to the GNP and graphite, the 002 basal plane of Mag‐GNP has shown a slight shift to the lower Bragg angle, as shown in Figure 1b. This suggests that the lattice *d*‐spacing has increased along the *c*‐axis, which could have an impact on *L*_c_ (crystallite thickness perpendicular to the layers along *c*‐axis). The greater interfacial distance implies a bigger crystal dimension along the *c*‐axis for Mag‐GNP in comparison with GNP and graphite. This indicates that Mag‐GNP has more capability to intercalate Na^+^ ions.^[^ ^38^ ^]^

In Figure 1a, Mag‐GNP shows the corresponding peaks of both Fe_3_O_4_ and GNP, exhibiting the Mag‐GNP as the composite structure of both Fe_3_O_4_ and GNP. It is challenging to distinguish maghemite from magnetite solely based on the XRD results. To gain clarity on the current lattice structure, we utilized FTIR. Figure 1c shows the FTIR spectra of graphite, Fe_3_O_4_, Mag‐GNP, and GNP. The spectra were obtained in the range of 500–3700 cm^−1^ and contain various vibrational bands. The peak at 580 cm^−1^ is due to the stretching vibration of the Fe—O bonds in magnetite,^[^ ^39^ ^]^ which helps differentiate it from maghemite,^[^ ^40^ ^]^ and this band, 580 cm^−1^, persists in both Fe_3_O_4_ and Mag‐GNP. Moreover, there are large number of oxygen‐containing functional groups in GNP, such as C=O (alkoxy) at 1061 cm^−1^;^[^ ^41^ ^]^ C=O (carbonyl) at 1730 cm^−1^;^[^ ^41^ ^]^ C—O (carboxy) at 1403 cm^−1^; C—O (epoxy) at 1227–1295 cm^−1^;^[^ ^16^ ^]^ and C=C (aromatics) at 1621 cm^−1^.^[^ ^42^ ^]^ It is worth noting that the alkoxy, epoxy, and carboxyl functional groups are significantly weaker in Mag‐GNP. This suggests that Mag‐GNP is a composite of Fe_3_O_4_ and a more reduced state of GNP. The graphite structure remained exposed while the magnetite nanoparticles were deposited on the GNP surface, as evidenced by the C—H stretching band at ≈3000 cm^−1^^[^ ^16^ ^]^ for both Mag‐GNP and GNP, with a slight shift to 2926 cm^−1^.^[^ ^43^ ^]^ The stretching bands at 1795 and 3425 cm^−1^ are ascribed to OH‐bending^[^ ^44^ ^]^ and OH‐stretching vibrations,^[^ ^45^ ^]^ respectively.

Raman spectroscopy is crucial in the characterization of nanocomposites of graphene and its derivatives based on structural features such as disorders^[^ ^46^ ^]^ and composition.^[^ ^47^ ^]^ As can be seen in Figure 1d, The Raman spectra of graphite display a dominant G peak at 1570 cm^−1^, which is assigned to the first‐order scattering of the E_2g_ mode.^[^ ^47^ ^]^ The GNP and Mag‐GNP demonstrate a marginal shift of their G peak toward 1585 cm^−1^, as a result of the functional groups connected to the graphite surface which are oxygenated and formed when electrochemical exfoliation occurs. These results are in agreement with the synthesis as explained above. The determination of the disorders is based on the intensity ratios observed in Figure 1d for the D and G bands. The intensity ratio of the D to G bands is 0.09, 0.97, and 0.73 for graphite, GNP, and Mag‐GNP, respectively. This suggests that the higher ratio of GNP compared to Mag‐GNP is due to a decrease in the average size of the *sp* ^2^ domains upon exfoliated GNP, which can be explained by the combination of graphitic domains with oxygen‐rich functional groups in GNP. This is because the Mag‐GNP particles are attracted to the cathode in electrochemical exfoliation due to the positive charge, and then they are reduced on the cathode, and Mag‐GNP is reduced when the exfoliation, particularly its graphitic structure, is reduced.

**Figure 2:**
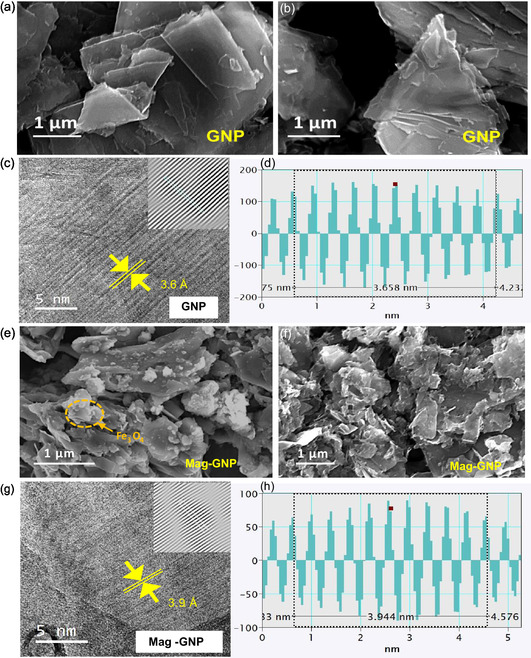
SEM images of a,b) the GNP and e,f) Mag‐GNP, and high‐resolution TEM images of c,d) the GNP and g,h) the Mag‐GNP. The inset of the c,g) is the inverse fast Fourier transform patterns, and the d,h) are the interlayer *d*‐spacing images of corresponding samples.

The surface and structural morphology of the samples were explored by SEM and TEM (**Figure** **2**). The SEM images of GNP depicted the platelets are sized around a few microns and their intact layers (Figure 2a,b). Figure 2b depicts the intact graphite structure with continuously separated graphite layers substantially preserved. During the electrochemical exfoliation process, the OH^−^ ions generated from the H_2_O functioned as nucleophiles and targeted the graphite edge planes containing SP_2_‐hybridized carbon atoms, resulting in a slightly widened interlayer distance (*d*_GNP_).^[^ ^48^, ^49^ ^]^ This modification was exhibited in the corresponding high resolution transmission electron miscrocopy image (Figure 2c). The *d*‐spacing images (Figure 2c,d) evidenced the *d*_GNP_ is 3.6 Å. The interlayer distance of graphite (*d*_graphite_) 3.4 nm^[^ ^49^ ^]^ is changed here, and it increased up to 3.6 Å due to the abovementioned phenomena. However, even though GNP layers are exfoliated, *d*_GNP_ = 3.6 Å is not enough to intercalate Na^+^ ions in anode layers; the minimum 3.7 Å^[^ ^21^ ^]^ is needed to accommodate Na^+^ ion inside the layered structure.

In Figure 2e,f, the excess Fe_3_O_4_ particles around 0.5–2 μm size can be seen on the surface of the GNP layers, as verified by energy dispersive X‐ray (EDX) elemental mapping on Figure S7 (Supporting Information). Upon introducing the Fe^2+^/Fe^3+^ ion combination into the electrochemical exfoliation process, the Fe^2+^/Fe^3+^ ions reacted with OH¯ ions forming the hydroxides. The hydroxides undergo a conversion process to form Fe_3_O_4_ inside the exfoliated graphene layers.^[^ ^47^ ^]^ This provides a wider interlayer distance (*d*_Mag‐GNP_) than *d*_GNP_. According to Figure 2h, *d*‐spacing image, 3.9 Å of *d*_Mag‐GNP_ is obtained, which is enough to accommodate the Na^+^ in‐between graphene layers.

**Figure 3:**
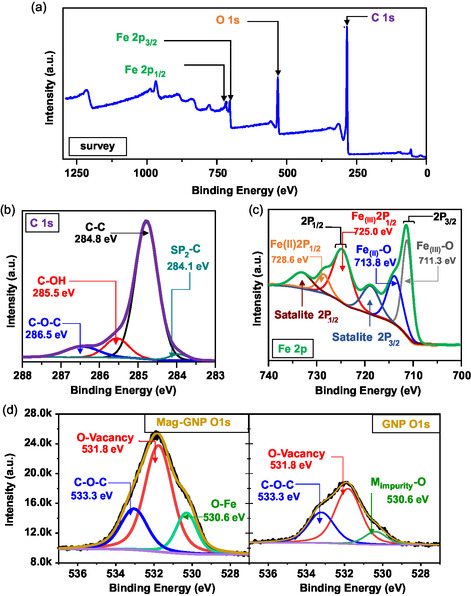
a) XPS survey spectrum of Mag‐GNP and their high‐resolution spectra representing b) C 1s, c) Fe 2p of Mag‐GNP, and d) comparison of O 1s spectrum of Mag‐GNP with GNP.

To further investigate the chemical composition of the synthesized anode materials, the Mag‐GNP was characterized using XPS. The survey spectrum (**Figure** **3**a) indicates that the composite is mainly composed of C, Fe, and O, while the presence of O could be ascribed to the oxidization and absorption of C of the GNP surface by the atmospheric oxygen and ascribed to the Fe_3_O_4_ nanoparticles. The high‐resolution XPS spectra of the C 1s are shown in Figure 3b. Due to electroexfoliation, functional groups are bonded to the surface of GNP's carbon skeleton. The GNP surface exhibits peaks at 285.5 eV (C—OH) and 286.5 eV (C—O—C).^[^ ^50^ ^]^ The Fe 2p_3/2_ signal shows two peaks at binding energies of 713.7 and 711.3 eV, indicating the bonding of Fe^2+^ and Fe^3+^ with oxygen, respectively (Figure 3c).^[^ ^23^ ^]^ The Fe 2P_1/2_ and Fe 2P_3/2_ satellite peaks are observed at 733.4 and 719 eV, respectively.^[^ ^51^ ^]^ A shift of 0.8 eV to higher binding energy occurs in the Fe 2p spectrum due to the bonding of Fe^2+^/Fe^3+^ with O or other functional groups that may form during electroexfoliation.^[^ ^52^ ^]^ The Fe—O bond at 530.6 eV and the O—H bond at 531.9 eV indicate the magnetite particle and activated functional groups in the surface GNP skeleton of the Mag‐GNP, respectively.^[^ ^53^ ^]^ Figure 3d displays the O 1s spectrum of GNP and Mag‐GNP spectrums, both of which exhibit the C—O—C ether peak at 533.3 eV. This aligns with the C—O—C ether peak displayed at 286.5 eV in the C 1s spectrum depicted in Figure 3b.^[^ ^50^ ^]^ Such findings suggest the emergence of functional groups on the surface of graphite resulting from the electroexfoliation of Mag‐GNP. In particular, the peak of oxygen vacancies at 531.8 eV^[^ ^54^ ^]^ in Mag‐GNP is larger than GNP, indicating that Mag‐GNP has a higher concentration of O vacancies. Additionally, GNP has shown a small percentage of metal oxide (M_impurity_–O) peak, shown in Figure 3d, at 530.6 eV^[^ ^54^ ^]^ due to impurities of the minerals in the bare vein graphite.^[^ ^55^ ^]^ The observation of the presence of higher oxygen vacancies in the Mag‐GNP structure coincides with the lower D/G ratio in the graphitic structure implying the partial reduction of the oxidized edge sites in the carbon skeleton. Further, the presence of oxygen vacant sites in the Fe_3_O_4_ structure also contributes to the presence of high oxygen vacancies. As our previous studies concluded, the presence of point defect/extended defect sites in the imperfect crystal structure of transition metal oxides gives rise to oxygen vacancies, and it helps to achieve superior electrochemical performances in energy storage devices such as Li–air batteries^[^ ^56^ ^]^ and hybrid supercapacitors,^[^ ^57^ ^]^ and similar support could expect here by providing a higher pseudocapacitance, in addition to intercalation of Na^+^ ions. Additionally, the previous reports identified these oxygen vacancies and heterostructures in Mag‐GNP‐enhanced reversible capacity, accelerated redox kinetics, and stable cycling life for sodium ion storage.^[^ ^24^ ^]^

**Figure 4:**
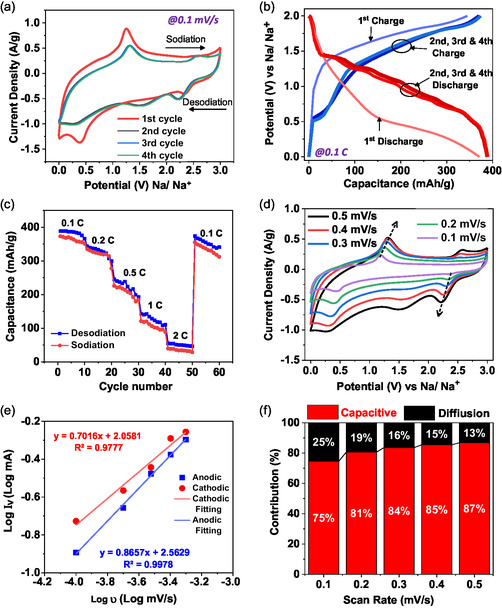
The consecutive first, second, and third cycles’ a) CV curves and b) charge–discharge profiles of the Mag‐GNP electrode in the voltage range of 0.01–3 V (vs Na/Na^+^), c) rate capability profiles of Mag‐GNP anode, d) CV curves e) log i–log *υ* plots of the Mag‐GNP composite at different scan rates, and f) contribution of Na^+^ ion storage mechanism of assembled SIB of Mag‐GNP.

To investigate the Na^+^ storage electrochemical properties of the Mag‐GNP, we fabricated the coin‐type (CR 2032) half cells with Na alloy as a counter and reference electrode. **Figure** **4**a exhibits the typical CV curve of the half‐cell of Mag‐GNP in 1 M NaPF_6_ electrolyte under 0 to 3 V of potential window and 0.1 mV s^−1^ of scan rate. The CV curve of the first cycle notably diverges from those of subsequent second, third, and fourth cycles, particularly during the discharge phase. During the initial discharge cycle, a higher intense peak is seen at ≈0.5 V (vs Na/Na^+^). This is generally attributed to the reaction between the electrode surfaces and interfaces leading to the formation of the SEI layer, which is a partial irreversible reaction between Mag‐GNP and electrolyte.^[^ ^58^, ^59^ ^]^ The presence of stable CV profiles in second to fourth consecutive cycles overlapping each other with sharp redox peaks indicates the highly reversible sodiation/desodiation reactions in the Mag‐GNP anode. During the CV study, peaks associated with the following conversion process (Equation (2) and (3)), as well as the intercalation reaction (Equation (4)) of Na^+^ ions in the Mag‐GNP anode, are expected to occur as explained below.^[^ ^31^ ^]^

(2) $${\text{Fe}}_{3}O_{4}+\;x{\text{Na}}^{+}+\;xe^{-}↔{\text{ Na}}_{x}{\text{Fe}}_{3}O_{4}$$

(3) $${\text{Na}}_{x}{\text{Fe}}_{3}O_{4}+\;\left(8-x\right){\text{Na}}^{+}+\;\left(8-x\right)e^{-}↔\;4{\text{Na}}_{2}\text{O }+\;3\text{Fe}$$

(4) $$6C+{\text{ Na}}^{+}+\;e^{-}↔{\text{ NaC}}_{6}$$

From the first cycle onward, the peaks at 1.5 and 0.5 V in the cathodic scan denote the forward reaction in Equation (2) and (3), respectively, where Fe^2+^/Fe^3+^ converted/reduced into their metallic states and the formation of Na_2_O also occurred.^[^ ^31^ ^]^ The oxidation of the formed Fe into Fe^2+^/Fe^3+^ could be ascribed to the sharp peak at 1.25 V on the anodic scan.^[^ ^60^ ^]^ Further, the respective cathodic and anodic peaks present at 2.3 and 2.5 V confirm the hybrid anode structure facilitates the swift intercalation and the deintercalation of Na^+^ into the expanded graphitic structure as denoted in Equation (4).^[^ ^61^ ^]^ These redox peaks in the CV curves are in good agreement with the charge–discharge profiles in Figure 4b measured in 0.1 C rate.

As Figure 4b demonstrates, the first discharge capacity of the Mag‐GNP anode is 352.6 mAh g^−1^ and gradually increased its maximum discharge capacitance to 399.7 mAh g^−1^ in the third cycle. Figure 4c illustrates the cycles in the multiple C rates such as 0.1, 0.2, 0.5, 1, and 2 C; the obtained averaged reversible capacitance is 397.6, 337.8, 228.2, 138.1, and 50.7 mAh g^−1^, respectively. To further inspect the redox kinetics of the Mag‐GNP anode, the half‐cell runs through CV measurements with multiple scan rates of 0.1–0.5 mV s^−1^ (Figure 4d). These CV curves exhibited the growing cyclic voltammograms exhibit developing overpotential with the scan rates indicating the quasireversible reactions.^[^ ^4^ ^]^ Additionally, the CV curves of GNP, RGO, and graphite anodes are exhibited in Figure S2 (Supporting Information). Factors influencing the Na^+^ storage in the different anode materials were studied to provide a deeper understanding of the Na^+^ ion storage mechanism, which generally categorized into the diffusion‐limited component and the capacitive components arise from the pseudocapacitance occur from the reaction with magnetite and from the surface‐oriented capacitive components occur from the defect sites on the surface.^[^ ^62^ ^]^ The simplified Randles–Sevcik equation (Equation (5)) demonstrates the correlation between peak current (*i*_p_) and scan rate (*υ*) where *C*_dl_ is the double‐layer capacitance.^[^ ^63^ ^]^

(5) $$i_{P}=AC_{\text{dl}}\upsilon$$

Using Equation (5), the electrochemical characteristics of the redox reactions that occurred on the electrode can be identified by analyzing the slope of log *i*_p_ and log *υ* plot in Figure 4e. The Mag‐GNP anode displayed the slopes 0.70 and 0.86 for cathodic and anodic reactions (Figure 4e), indicating that the electrochemical reaction is controlled by both Na^+^ diffusion and capacitive contribution. As indicated in Figure 4f, 3/4th of the total capacitance is contributed by the capacitive component during the 0.1 mV s^−1^ scan. However, the total capacitance and the capacitive component are both lower in the other studied anode materials as depicted in Figure S4 (Supporting Information). Hence, the study demonstrates a substantial contribution in magnetite and the expanded graphitic network in Mag GNP toward achieving substantial Na ion intercalation and conversion. Figure S3 (Supporting Information) demonstrates the variation in capacitive and diffusive components as the scan rate increases. Even though the diffusion was limited during the faster scans, it is noteworthy to mention that the sizable contribution of 13% was made by the diffusion toward total capacitance at 0.5 mV s^−1^, showcasing the superiority of the hybrid anode. This high contribution of Mag‐GNP is ascribed to the larger interlayer distance, lower Na^+^ diffusion barrier, and higher electronic conductivity, facilitating swift sodiation and desodiation.

Inside the Mag‐GNP, the Fe_3_O_4_ provides a high pseudocapacitive nature and its increase in the *d*‐spacing (*d*_GNP_) of the graphene layers enhanced the excellent electrical conductivity and provided structural stability under electrochemical reactions. To illustrate the high performance of Mag‐GNP as an anode material, we compare the electrochemical properties of Mag‐GNP with those of conventional anode materials such as graphite, RGO, and GNP. For this comparison, synthesized RGO^[^ ^64^ ^]^ was fabricated on Cu foil and assembled into CR‐2032 cells with Na alloy as a counter and reference electrode.

**Figure 5:**
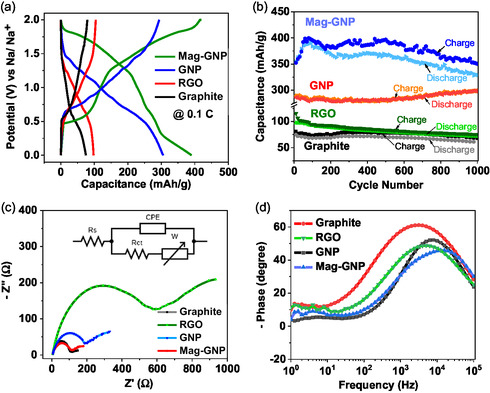
a) The comparison of galvanostatic charge–discharge curves, b) cyclability, c) Nyquist plot, and d) bode plot of Mag‐GNP with other conventional graphene derivative anode material: graphite, RGO, and GNP.

**Figure** **5**a shows the galvanostatic charge–discharge curves of graphite, RGO, GNP, and Mag‐GNP at a rate of 0.1 C. Their discharge and charge‐specific capacities for graphite are 74.8 and 79.3 mAh g^−1^, respectively. The capacitance of graphite is low because the Na^+^ ions struggle to intercalate between the graphite layers due to not enough space between them.^[^ ^65^ ^]^

The RGO has shown 98 and 104.3 mAh g^−1^ for their discharge and charge capacitance. This may, due to the RGO's poor reduction step, decrease the electrical conductivity. GNP had higher discharge and charge capacitance values (305.4 and 283.8 mAh g^−1^) compared to graphite, indicating better performance in intercalating Na^+^ ions due to wider interlayer distance in GNP (3.6 Å). Significantly, the Mag‐GNP has shown the obtained highest discharge and charge capacitance 390 and 420 mAh g^−1^ attributed to their wide *d*‐spacing distance as well as the faradic nature of the magnetite nanoparticles. This demonstrates that the magnetite particles enhance the *d*‐spacing distance in Mag‐GNP nanocomposite. The shapes of the charge–discharge curves of graphite, RGO, and GNP are nearly the same because they only undergo Na^+^ ion intercalation. However, Mag‐GNP has shown the different shapes of their charge–discharge curves compared to the graphite, RGO, and GNP because of the Na^+^ ion intercalation and the additional faradic reaction of Na^+^ ions with Fe_3_O_4_.

Around the 100 cycles, the average discharge capacitance obtained was 351.7 mAh g^−1^ in Mag‐GNP (Figure 5b). The total capacitive retention after 1000 cycles was 99.7% compared to the first cycle and Coulombic efficiency retained around 96%. The coin cells were further experimented with for the cycling process for charge /discharge in different C rates to identify their rate capabilities. At the lowest C rate (0.1 C), the Mag‐GNP anode shows a capacitance of 398.5 mAh g^−1^, which is 5 times higher than that of the commercial graphite (74.8 mAh g^−1^) and around 4 times higher than the RGO (102.3 mAh g^−1^) and significantly improved than GNP (286.1 mAh g^−1^). The cycling Coulombic efficiency and corresponding charge–discharge profiles of the graphite, RGO, GNP, and Mag‐GNP are shown in Figure S4 (Supporting Information), respectively. The inability to provide the full reversibility by the conversion‐type Na^+^ storage mechanism in magnetite may account for the reduced Coulombic efficiency in the Mag‐GNP anode‐based SIB. In comparison, GNP anode‐based SIB operated with nearly 100% efficiency, and the capacitance gradually increased as the material was further activated due to the layered architecture of GNP facilitates electrolyte penetrations and provides sites for Na^+^ ion storage in the progressive cycling process (Figure 5b). Certain instabilities and mechanical strains occur on the SEI and the anode during the conversion‐type sodiation may be accountable for the observed capacity fluctuations in the Mag‐GNP anode‐based SIB. However, despite the minor fluctuations, the hybrid architecture was able to provide superior performances overcoming the negative effects occurring by the volume expansion during the conversion‐type charge storage during the conversion‐type Na^+^ storage on charging may be accountable for the observed capacity fluctuations in the Mag‐GNP anode‐based SIB. However, despite the minor fluctuations, the hybrid architecture was able to provide superior performances overcoming the negative effects caused by the volume expansion during the conversion‐type charge storage. These observations further verify the structural stability of the Mag‐GNP composite, where repeated reconstruction of the SEI consumes significant amounts of the electrolyte and Na^+^ ions, thereby lowering the Coulombic efficiency and capacity retention in cases of structural collapse.^[^ ^66^ ^]^ The ICE of the Mag‐GNP, GNP, graphite, and RGO is 85, 82.5, 68.1, and 80%, respectively. From the second cycle, the Coulombic efficiency of the anodes was obtained as 99.7, 100.0, 97.0, and 94.3% for Mag‐GNP, GNP, graphite, and RGO, respectively (Figure S3, Supporting Information). This indicated that the Mag‐GNP and GNP have high ICE values compared to the RGO and graphite, demonstrating a high reversibility in sodiation and desodiation process.^[^ ^67^ ^]^

To understand the electrical conductivity parameters of the synthesized materials, EIS measurements were carried out for each material as shown in Figure 5c,d. The Nyquist plot in Figure 5c depicted identical solution resistance from all anode materials, attributed to high electrical contact between electrolyte and electrode. Previous studies suggest that the diffusion of metal ions is related to the phase angle in the low‐frequency range. The faster the Na^+^ ions diffusion, the smaller the phase angle.^[^ ^68^ ^]^ The charge‐transfer resistance (*R*_ct_) values we obtained from the EIS study (Figure 5c) are 110, 115, 200 Ω, and around 600 Ω for graphite, Mag‐GNP, GNP and RGO, respectively. As *R*_ct_ denoted from the semicircle of the Nyquist plot Figure 5c, the RGO has shown the highest value due to the poor reduction process. However, as explained in our modified electrochemical exfoliation, the GNP has some functional groups that affected the continuity of the π‐electron clouds. Notably, Mag‐GNP has a more reduced nature than the GNP, which agrees with the low *R*_ct_ values. In addition, the Mag‐GNP's high interlayer distance of 3.9 A provided a more hollow nature to Mag‐GNP that provided fast metal ion transportation kinetics.^[^ ^57^ ^]^ Even though there is no ample space for Na^+^ ion in graphite, the naturally occurred π‐electron cloud provides the high electrical conductivity for graphite denoted by the lowest *R*_ct_.

As exhibited in Figure 5d, Mag‐GNP and GNP have considerably smaller phase angles in the low‐frequency range (1–10 Hz) compared to the RGO and graphite. Among them, Mag‐GNP has shown the smallest phase angle 3.2°, indicating the highest Na^+^ ion mobility inside the materials. GNP, RGO, and graphite have shown their smallest phase angle in the range of 1–10 Hz is 4.1°, 10.0°, and 10.9°, respectively. The diffusion coefficient of Na^+^ during the initial sodiation and desodiation was determined using the GITT. This measurement can provide insights into the Na^+^ diffusion kinetics in the Mag‐GNP. Utilizing Fick's second law of diffusion, the diffusion coefficient of Na^+^ in the electrode is calculated based on Equation (6) as follows:^[^ ^69^ ^]^

(6) $$D_{\text{Na}}^{+}=\frac{4}{\pi \tau }{\left[\frac{m_{b}V_{M}}{M_{b}S}\right]}^{2}{\left[\frac{\Delta E_{s}}{\Delta E_{\tau }}\right]}^{2}$$

In this context, *τ* signifies the duration of the applied galvanostatic pulse. The term *m*_*b*_ represents the active mass at the anode, while *V*_*M*_ and *M*_*b*_ denote the molar volume and molar mass of the active material, respectively. *S* refers to the geometrical area of the electrode. Furthermore, Δ*E*_*s*_ and Δ*E*_*τ*_ are derived from the GITT curve, as depicted in Figure S6 (Supporting Information).

The GITT was utilized to demonstrate the enhancement in ionic conductivity of the Mag‐GNP electrode in comparison with the GNP electrode. Throughout the charge and discharge processes, the diffusivity of Na^+^ in Mag‐GNP was found to be superior to that in bare GNP, as illustrated in Figure S7 (Supporting Information). The Mag‐GNP has a greater interlayer distance, measuring 3.9 Å, compared to the bare GNP, which has an interlayer distance of 3.6 Å. This difference is considered the primary reason why Mag‐GNP shows superior electrochemical performance and greater stability during long‐term cycling compared to GNP. The prompt decrease in the D_Na_^+^ value around 0.1 to 0.05 V is attributed to the slow dynamics of Na^+^ intercalation into the graphite interlayer.^[^ ^69^, ^70^ ^]^ In contrast, the recovery of D_Na_^+^ values near the cutoff voltage is due to the adsorption and aggregation of Na^+^ within the closed nanopores. It is noteworthy that the average diffusion coefficient for Na^+^ in the plateau region in (0.1–0.05 V) has been calculated to be 3.031 × 10^−11^ and 3.661 × 10^−11^ cm^2^ s^−1^ for charging and discharging process in Mag‐GNP and 4.026 × 10^−12^ and 1.774 × 10^−12^ cm^2^ s^−1^ for charging and discharging process in GNP, respectively. These results are consistent with their corresponding rate performance and suggest the presence of Magnetite enhancing the interlayer distance of GNP, thereby enhancing Na^+^ storage capability in low‐voltage regions.

**Figure 6:**
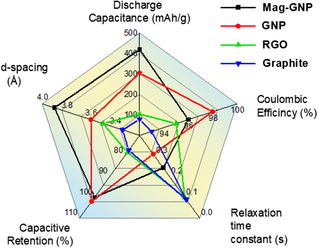
Radar chart of discharge capacitance of anodes (from half‐cell setup of anode active materials), Coulombic efficiency, relaxation time constant, capacitive retention (at 1000 cycles), and interlayer distance (*d*‐spacing) of Mag‐GNP, GNP, RGO, and graphite.

To describe the correlation of electrochemical performance of the different active materials, the discharge capacitance of anodes, Coulombic efficiency, relaxation time constant, capacitive retention, and interlayer distance are presented in the schematic radar graph in **Figure** **6**. It is worth noticing that the evaluation of Mag‐GNP from the bare vein graphite is quite extraordinary. The *d*‐spacing obtained is 3.9, 3.6, 3.5, and 3.34 Å corresponding to Mag‐GNP, GNP, RGO, and graphite, respectively. This high *d*‐spacing of 3.9 Å allows intercalation of Na ions inside the layers of GNP which is correlated with its 420 mAh g^−1^ of the highest discharge capacitance and 99.9% high Coulombic efficiency. However, the electron cloud structure of graphite was a little bit depleted with GNP, RGO, and Mag‐GNP. Therefore, graphite shows the lowest relaxation time constant 0.09 s, and then RGO, Mag‐GNP, and GNP show 0.1, 0.243, and 0.31 s, respectively. The Mag‐GNP's graphitic parts were more reduced compared to the GNP; therefore, Mag‐GNP has high electrical conductivity which leads to lower relaxation time in contrast to the GNP. In comparison, the superior electrochemical performance of Mag‐GNP is attributed to its high *d*‐spacing and high electrical conductivity (**Table** **1**).

**Table 1:** Comparison of electrochemical performance and morphological parameters of reported anode materials with obtained results.

Anode material	Discharge capacitance [mAh g^−1^]	Capacitive retention [%]	Coulombic efficiency [%]	Number of cycles	*d*‐space [Å]	Particle shape and size [nm μm^−1^]	References
Hollow carbon nanowires	251	82.2	98	400	3.7	Nanotubes, 150 nm	[12]
FeSe_2_ carbon cuboids	331	82	100	1000	2.9	Cube, 1 μm	[20]
Expanded graphite (EG)	284	73.9	100	2000	4.3	2D sheets, n.a.	[10]
Tailor‐made carbon materials with hierarchical porosity	130	99.8	97.9	125	n.a.	Macro/mesopores system, 2–4 μm	[13]
Tailored graphite anodes	120	93	99.9	1000	11.6–12.5	Omni shape particles, 10 μm	[3]
Porous FeP/C composite nanofibers	760	98.6	99.6	1000	1.6	Nanofibers, 50–100 nm	[5]
VO_2_ nanobelts with nitrogen‐doped carbon nanosheets	258	88.4	99.9	1800	2.1	Nanobelts, 1–5 μm	[21]
RGO	131.7 (at 1.8 A g^−1^)	87.7 (at 400 mA g^−1^)	n.a.	900	3.8	RGO with a long‐range‐ordered layered thin wrinkled structure with nano cavities and nano holes	[73]
Thermally processed electrochemical graphite oxide	163 (at 0.5 A g^−1^)	60.84	100	2000	6.2	Multilayered graphite oxide crystalline lattice with 6.2 Å *d*‐space	[74]
Thermally treated expanded graphite material employing MoS_x_ pillars (EGMoS_x_)	281 (at 0.1 A g^−1^)	77.5	97.2	100	5.4	Amorphous expanded graphite material employing MoS_x_ pillars	[75]
Hard carbon spheres encapsulated with graphene networks	122 (at 10 A g^−1^)	87.1	100	4000	3.7	Hard carbon spheres encapsulated with graphene networks Of 5 nm thickness	[76]
Red phosphorous incorporated expanded graphite	296 (at 0.5 A g^−1^)	83.3	100	1000	3.8	Multilayered expanded graphite incorporated with red phosphorous	[77]
Mag‐GNP	420	99.9	96	1000	3.9	Multilayered, 500–1000 nm	This work

n.a.: not applicable.

It must be mentioned that our Mag‐GNP reported in this article does not have the highest discharge capacitance values for anode material for SIBs but it shows a remarkable level of performance, 420 mAh g^−1^. However, as mentioned in the literature, the minimum *d*‐space is 3.7 Å which provides the minimum space for intercalating the Na^+^ ion into graphitic layers achieved by only a few materials.^[^ ^3^, ^21^, ^71^ ^]^ Mag‐GNP demonstrates its outstanding performance by obtaining the interlayer spacing of 3.9 Å (*d*_Mag‐GNP_) which is higher than the minimum level. Compared to other graphitic materials such as expanded graphite, hollow carbon nanowires, nanosheets, and carbon cuboids Mag‐GNP shows 96% Coulombic efficiency, which represents a high value indicating the high reversibility.^[^ ^72^ ^]^ The 99.9% capacitive retention with over 1000 cycles which is consistent with the results reported in the literature, indicating the applicability of Mag‐GNP as an anode material for SIBs. There is room to improve the Coulombic efficiency and electrochemical performance by changing the size of the particles and the content of Fe_3_O_4_ with GNP. We hope to try to optimize these changes with magnetite and GNP complexes in the forthcoming studies. These composites are useful in energy storage technologies such as LIB anodes, supercapacitors, and other applications such as photocatalysts and optoelectronic devices.^[^ ^47^ ^]^ Further, we invite theoretical experts to study the introduced hypothesis and we also try to validate our results through DFT calculations in the future.

## 4 Conclusion

In summary, magnetite nanoparticles embedded in GNP (Mag‐GNP) have been successfully synthesized using modified electrochemical exfoliation with the assistance of Fe^3+^ and Fe^2+^ ions. This composite exhibits a unique layered architecture with an increment of interlayer distance of 3.9 Å, which allows a shift intercalation of Na^+^ ions into the graphitic structure while the magnetite provides additional capacity with conversion type Na^+^ storage as the composite used as the SIB anode. As a result, this layered composite exhibits high electrochemical performance: 420 mAh g^−1^ of reversible discharge capacitance at 0.1 C while displaying 96% of Coulombic efficiency and 99.9% capacity retention under 1000 cycles. In comparison, GNP with 3.6 Å of interlayer distance only provides 305 mAh g^−1^ reversible discharge capacitance at 0.1 C. Our modified electrochemical exfoliation is a good methodology for increasing the interlayer distance of Mag‐GNP and GNP contact from 3.34 Å, which is the intrinsic interlayer distance of graphite. Kinetic and mechanistic studies conclusively demonstrate that the embedded magnetite nanoparticles significantly enhance the Na^+^ diffusion coefficient in the plateau region around 3.031 × 10^−11^ and 3.661 × 10^−11^ cm^2^ s^−1^ for charging and discharging process be readily improved compared to the 4.026 × 10^−12^ and 1.774 × 10^−12^ cm^2^ s^−1^ for charging and discharging process in GNP. These findings support the successful facile methodology to convert graphite anodes into high capacitive Na^+^ ion battery anodes exploiting the high interlayer distance and promising cycling performance for large‐scale energy storage applications.

## Conflict of Interest

The authors declare no conflict of interest.

## Author Contributions

**Rukshan Karunarathna**: conceptualization (equal), formal analysis, writting‐ original draft. **Harsha Ranasinghe Arachchige**: conceptualization (equal), formal analysis, writting‐ original draft. **Shadeepa Karunarathne**: conceptualization (equal), formal analysis, writing ‐review & editing. **W. Parakrama Sanjeewa Lakshitha Wijesinghe**: conceptualization (equal). **Chanaka Sandaruwan**: conceptualization (equal). **M. M. M. Prasanga Gayanath Mantilaka**: conceptualization (equal). **Yasun Y. Kannangara**: conceptualization (equal); supervision (equal). **Amr M. Abdelkader**: conceptualization (lead); formal analysis (supporting); funding acquisition (lead); resources (lead); supervision (lead); writing—review & editing (lead).

## Supporting information

Supplementary Material

## Data Availability

The data that support the findings of this study are available from the corresponding author upon reasonable request.
