# Supplementary material for: Intercalating Graphite‐Based Na‐Ion Battery Anodes with Integrated Magnetite
Source: Small Sci. 2024 Dec 19;5(2):2400405. doi: 10.1002/smsc.202400405 (PMC11934899; doi:10.1002/smsc.202400405)
Supplement: Supplementary file 1 — Supplementary Material [file SMSC-5-2400405-s001.pdf]

<u>**Supplementary Information**</u>

Intercalating Graphite-Based Na-Ion Battery Anodes with Integrated Magnetite

Rukshan Karunarathna [+ 1,2], Harsha Ranasinghe Arachchige [+ 1,3], Shadeepa Karunarathne [+4,] W.P.S.L. Wijesinghe [3], Chanaka Sandaruwan [3], M.M.M.G.P.G. Mantilaka [5], Yasun Y. Kannangara [*3,5] Amr M. Abdelkader. [*4]

[+] Authors are contributed equally.

[1] Postgraduate Institute of Science, University of Peradeniya, 20400, Sri Lanka.

[2] Centre for Nanodevices Fabrication and Characterization (CNFC), Faculty of Technology, Sabaragamuwa University of Sri Lanka, Belihuloya 70140, Sri Lanka.

[3] Sri Lanka Institute of Nanotechnology, Technology Park, Homagama, 10800, Sri Lanka.

[4] Faculty of Science and Technology, Bournemouth University, Talbot Campus, Fern Barrow, Poole, BH12 5BB, UK.

[5] QBITS Labs, Research and Development, Codegen International (Pvt) Ltd, Trace City, Maradana, Sri Lanka.

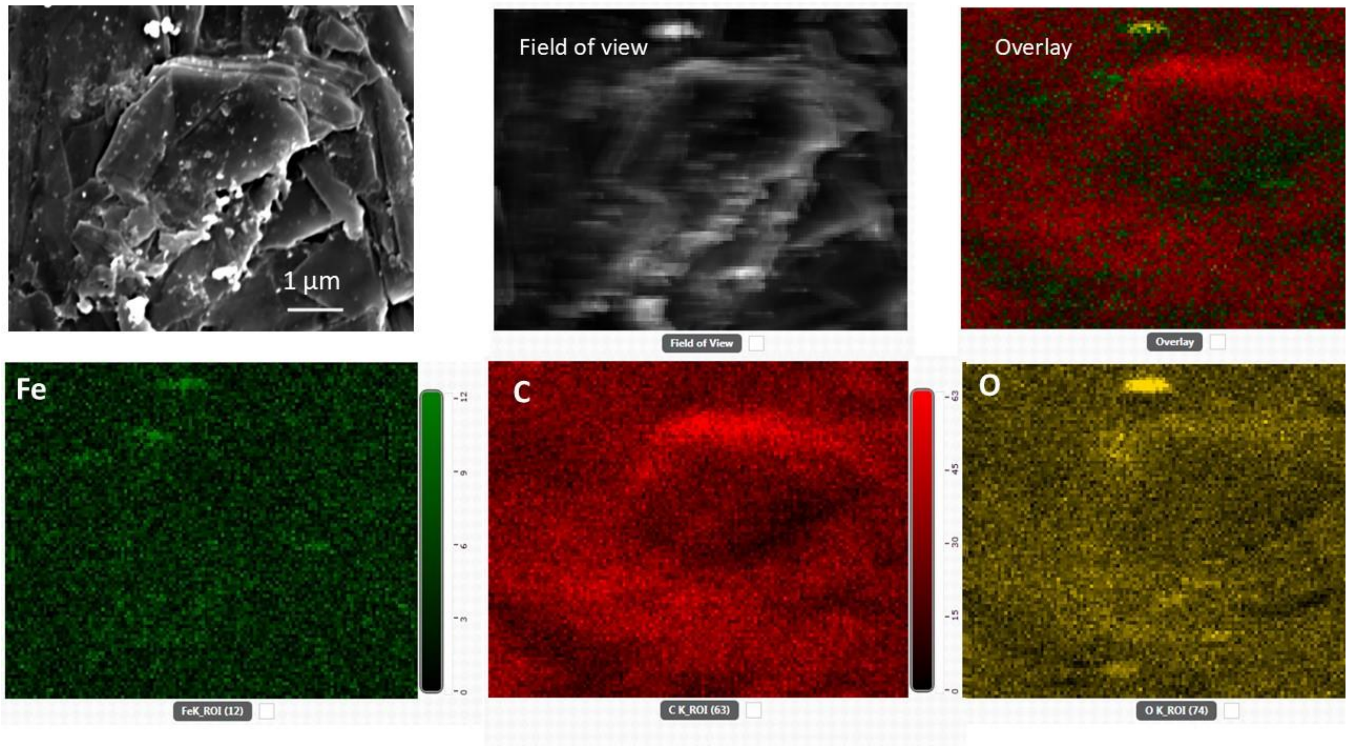

Fig. S1: EDX elemental mapping of the Mag-GNP hybrid structure

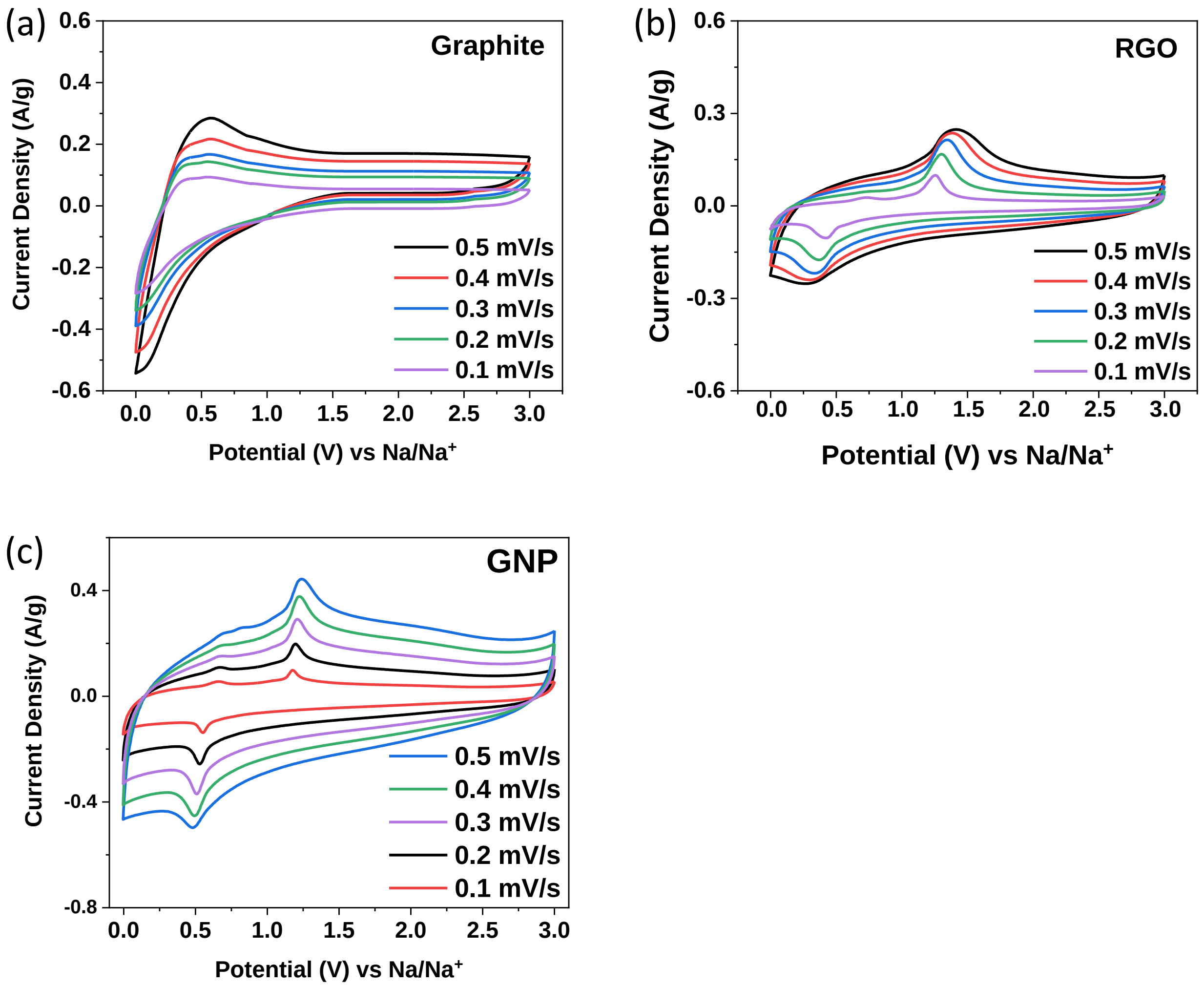

Fig. S2: Cyclic voltammetry of (a) Graphite, (b) RGO, and (c) GNP in different scan rates

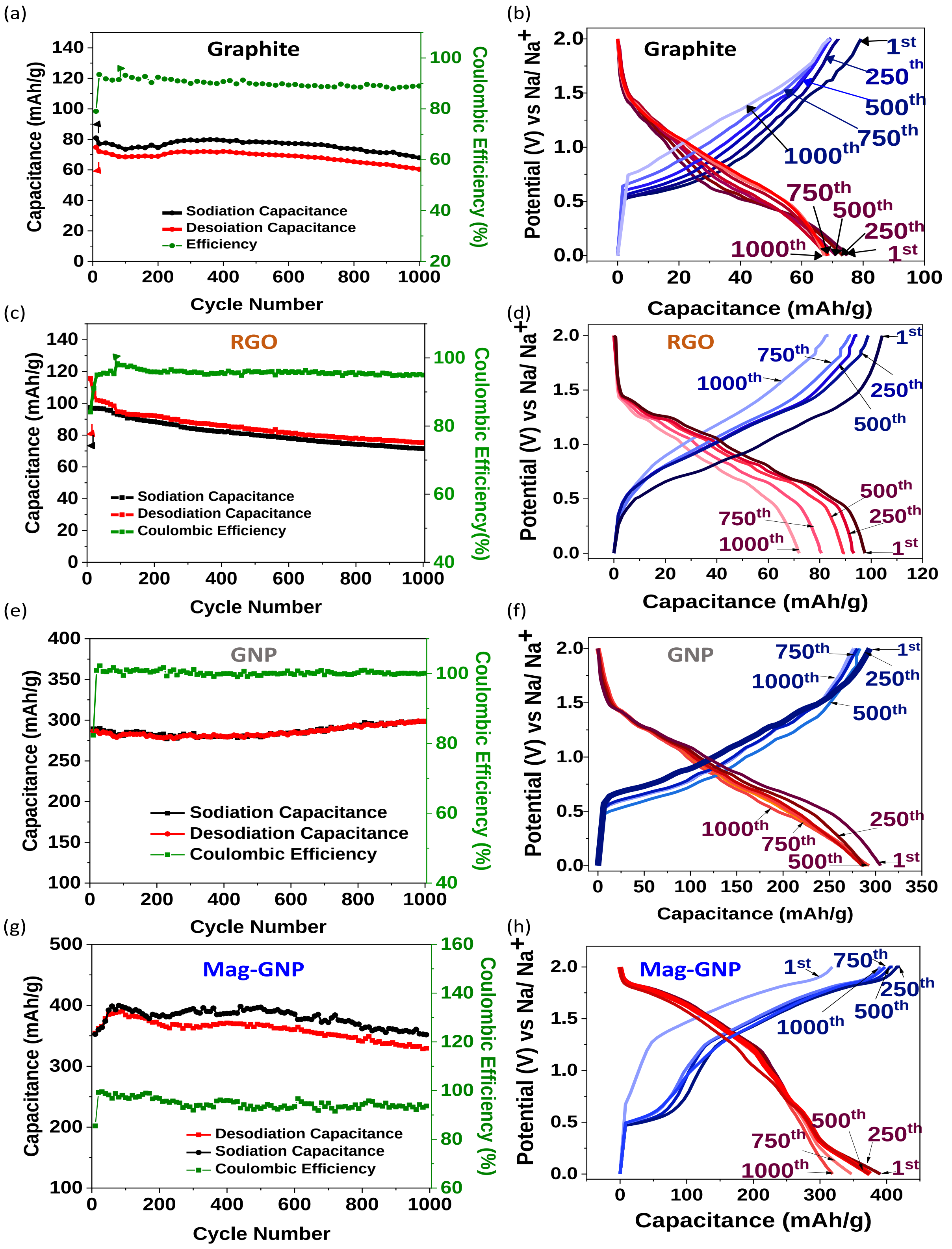

Fig.S3: Cyclability profiles of (a) Graphite (b) RGO (c) GNP and (d) Mag-GNP with their coulombic efficiencies and Galvanostatic charge-discharge curves of (b) Graphite (d) RGO (f) GNP and (h) Mag-GNP around 1000 cycles.

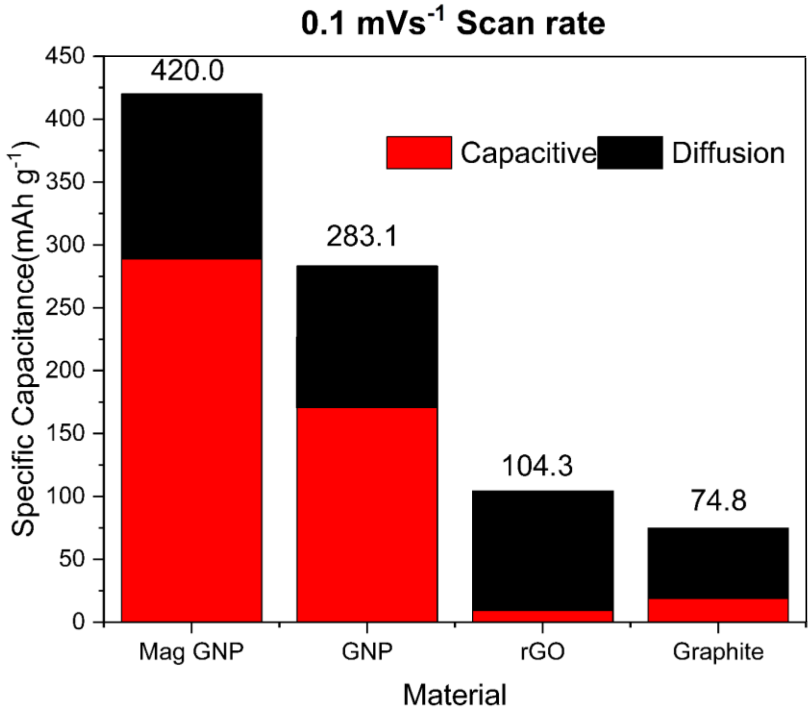

Fig.S4: Capacitive and Diffusive components of charging capacity at 0.1 mV/s for different materials.

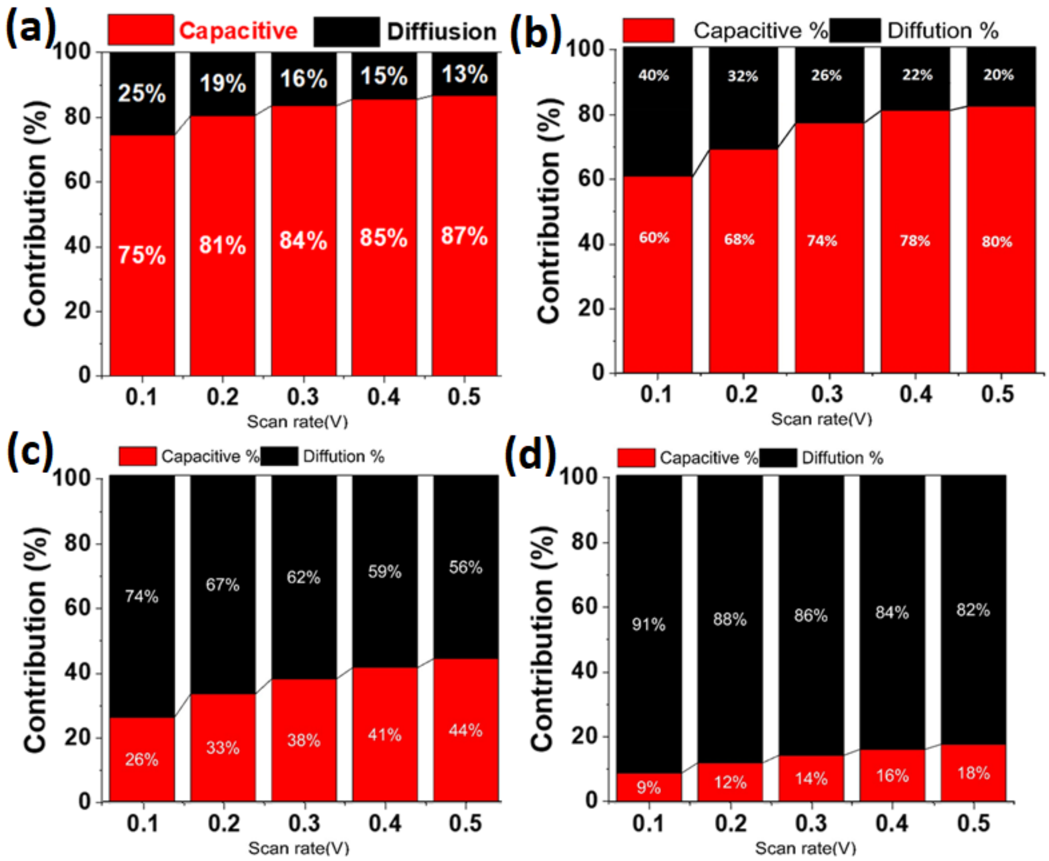

Fig.S5: Study of the Na^+ storage mechanism of the assembled SIBs with anodes of (a) Mag GNP, (b) GNP, (c) Graphite and (d) RGO under different scan rates.

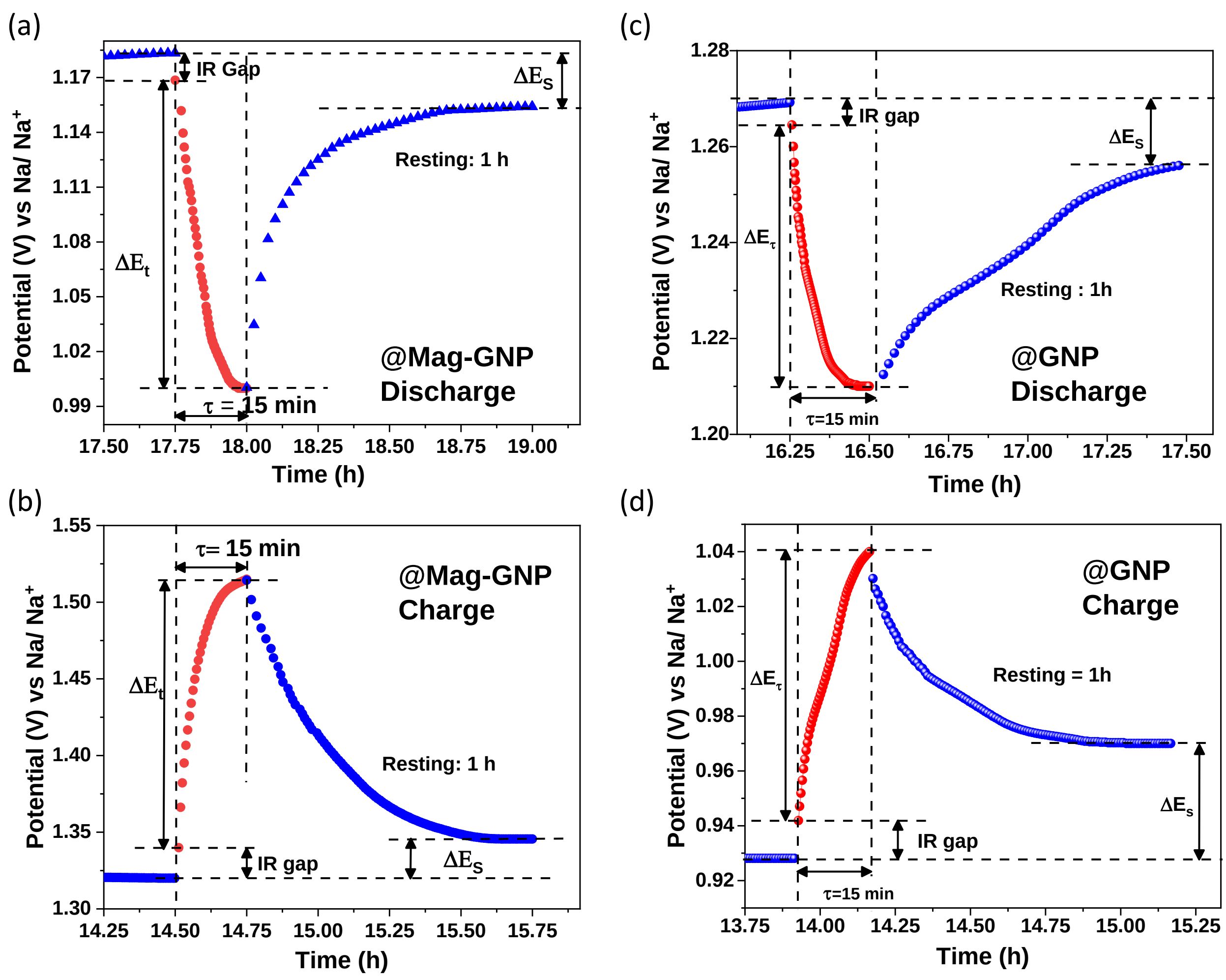

Fig. S6: Parameter curve of GITT during (a) discharging (b) charging, in Mag-GNP and (c) discharging (d) charging, in GNP, respectively.

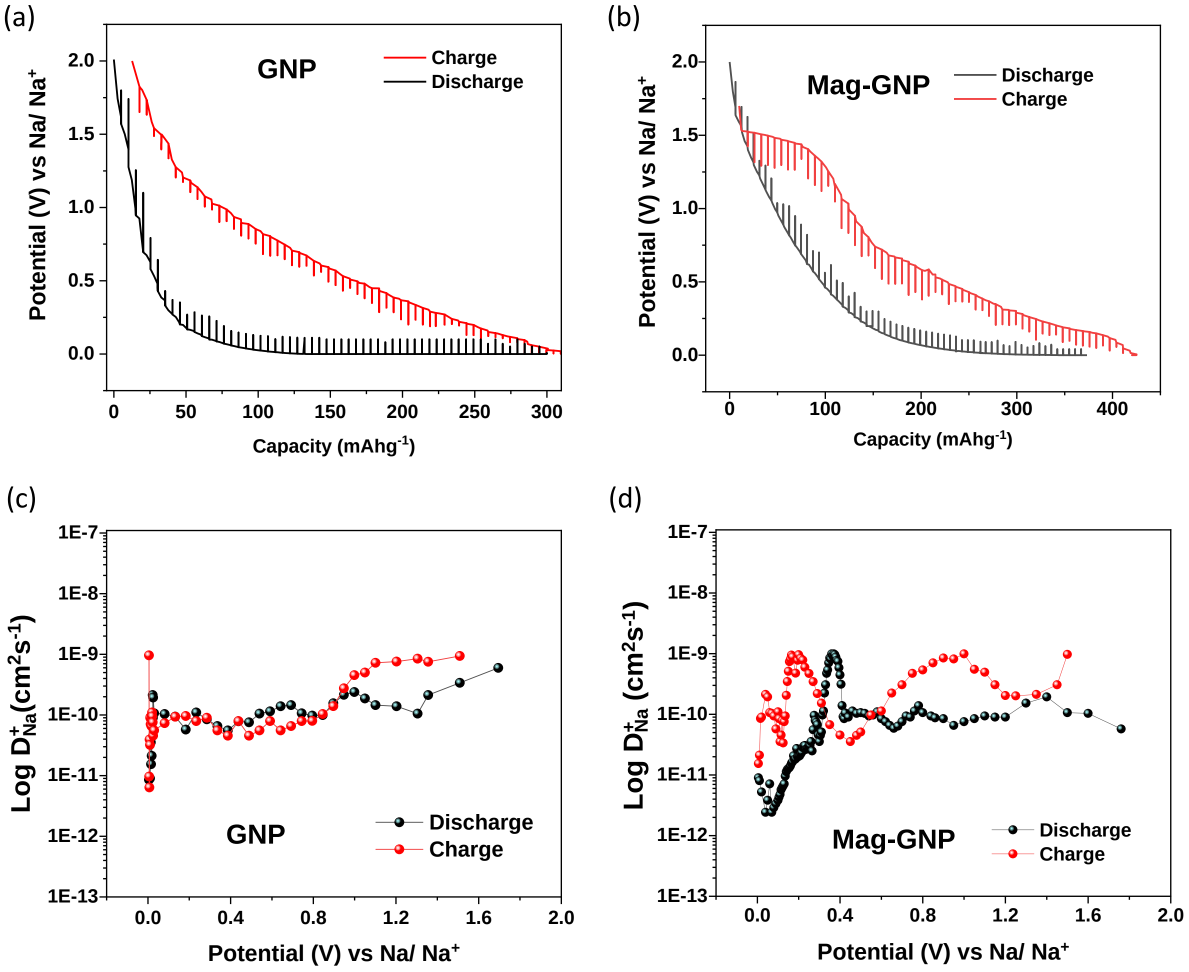

Fig. S7: GITT curve for both (a) GNP and (b) Mag-GNP in the potential range of 0.01–2.0 V at a charge/discharge rate of 0.2C for each pulse for 15 min and following relaxation for 1 hour, and calculated Na^+ diffusion coefficient of (c) GNP and (d) Mag-GNP.

.
